# Endophytic Fungi Isolated from Oil-Seed Crop *Jatropha curcas* Produces Oil and Exhibit Antifungal Activity

**DOI:** 10.1371/journal.pone.0056202

**Published:** 2013-02-08

**Authors:** Susheel Kumar, Nutan Kaushik

**Affiliations:** 1 TERI University, Vasant Kunj, New Delhi, India; 2 The Energy and Resources Institute (TERI), India Habitat Center, New Delhi, India; Pacific Northwest National Laboratory, United States of America

## Abstract

*Jatropha curcas* L., a perennial plant grown in tropics and subtropics is popularly known for its potential as biofuel. The plant is reported to survive under varying environmental conditions having tolerance to stress and an ability to manage pest and diseases. The plant was explored for its endophytic fungi for use in crop protection. Endophytic fungi were isolated from leaf of *Jatropha curcas*, collected from New Delhi, India. Four isolates were identified as *Colletotrichum truncatum*, and other isolates were identified as *Nigrospora oryzae*, *Fusarium proliferatum*, *Guignardia cammillae*, *Alternaria destruens*, and *Chaetomium* sp. Dual plate culture bioassays and bioactivity assays of solvent extracts of fungal mycelia showed that isolates of *Colletotrichum truncatum* were effective against plant pathogenic fungi *Fusarium oxysporum* and *Sclerotinia sclerotiorum*. Isolate EF13 had highest activity against *S. sclerotiorum*. Extracts of active endophytic fungi were prepared and tested against *S. sclerotiorum*. Ethyl acetate and methanol extract of *C. truncatum* EF10 showed 71.7% and 70% growth inhibition, respectively. Hexane extracts of *C. truncatum* isolates EF9, EF10, and EF13 yielded oil and the oil from EF10 was similar to oil of the host plant, i.e., *J. curcas*.

## Introduction

*Jatropha curcas* L. (Family: *Euphorbiaceae*), a promising energy crop, is being extensively studied for developing bio-fuel technology as well as for other beneficial use, viz., antimicrobial and pesticidal activity. Leaf extract of Jatropha has been known to possess insecticidal activity against mosquito larvae [1]. Insecticidal property of the seed oil and plant extract has been reported against cotton bollworm, pests of pulses, potato and corn [2]. Diseases of *J. curcas* include anthracnose caused by *Colletotrichum gloeosporioides* [3], black rot caused by *Botryosphaeria diplodea* [4], root rot caused by *Rhizoctonia bataticola* [5] and root rot and collar rot caused by *Lasiodiplodia theobromae* [6]. *J curcas* is widely distributed in many parts of tropics and subtropics of the world and can be easily cultivated in low to high rainfall areas of saline and marshy lands [7]. Its adaptation to diverse agro-climatic condition is linked to its ecological fitness, which possibly could be in part, due to the presence of endophytic fungi [8]. Endophytic fungi live inside the plant without causing any overt negative effect on the host, rather they protect the host plant from pests and diseases [9]. The ability of endophytic fungi of grasses to provide protection from insect herbivore [10], [11], [12] drew the attention of researchers to exploit the endophytic microflora, especially fungi for better health of crop plants. Later, several workers reported endophytic fungi from plants and their bio-activity against wide range of pests and pathogens. Antifungal activity of endophytic fungi is well documented [13], [14], [15], [16], [17]. With the hypothesis that pesticidal property in Jatropha extracts and its seed oil is in part due to the presence of endophytic fungi, the present study was undertaken to assess the antifungal activity of endophytic fungi present in *J. curcas* against *Rhizoctonia solani*, *Sclerotinia sclerotiorum*, and *Fusarium oxysporum* phytopathogens. These phytopathogens have a wide host range and cause major losses in important food crops like rice, maize, wheat, and chickpea.

## Results

### Isolation of endophytic fungi

Isolation of endophytic fungi was performed during June 2007 to August 2007. Nine endophytic fungi were isolated from 44 tissue segments of the leaf of *J. curcas* with isolation frequency of 20.5% and no endophytic fungus appeared out of 16 tissue segments of petiole kept for isolation up to 30 days. Pure cultures of the nine endophytic fungi EF8-EF16 were identified by rDNA sequencing of their ITS region [18]. Isolate EF8 was identified as *Nigrospora oryzae*, four isolates EF9, EF10, EF13, and EF 14 were identified as *Colletotrichum truncatum*, while EF11 was identified as *Fusarium proliferatum*, EF12 as *Chaetomium* sp., EF15 as *Guignardia camelliae*, and EF16 as *Alternaria destruens*. BLAST per cent similarity to sequences in the NCBI database from previously identified fungi ranged from 96% to 100% (Table 1).

**Table 1:** Identification of endophytic fungi isolated from *Jatropha curcas*, their per cent similarity in BLAST and their bio-efficacy against plant pathogens.

Isolate name	Near matching with	GenBank Accession No.	Per cent similarity	Activity against PP fungi		
				R	S	F
EF8	*Nigrospora oryzae* isolate MS-544	GQ176271	98	−	−	+
EF9	*Colletotrichum truncatum* isolate CT0524	GQ176272	100	−	++	+
EF10	*Colletotrichum truncatum* isolate CT0531	GQ176273	99	−	+++	++
EF11	*Fusarium proliferatum* isolate Z23-28	GQ176274	99	−	−	−
EF12	*Chaetomium* sp. INBI 2-26(-)	GQ176275	96	−	+	+
EF13	*Colletotrichum truncatum* isolate CT0531	GQ176276	99	−	+++	+
EF14	*Colletotrichum truncatum* isolate CT0531	GQ176277	99	−	+	++
EF15	*Guignardia camelliae* isolate T120	GQ176278	99	−	+	−
EF16	*Alternaria destruens* isolate Alt2	GQ176279	99	−	−	−

**PP fungi-** Plant Pathogenic fungi **F-** *Fusarium oxysporum*, **R-** *Rhizoctonia solani*, **S-** *Sclerotinia sclerotiorum*.

− Not active; + slightly active; ++ moderately active; +++highly active.

### Dual culture bioassay of endophytic fungi

**Figure 1:**
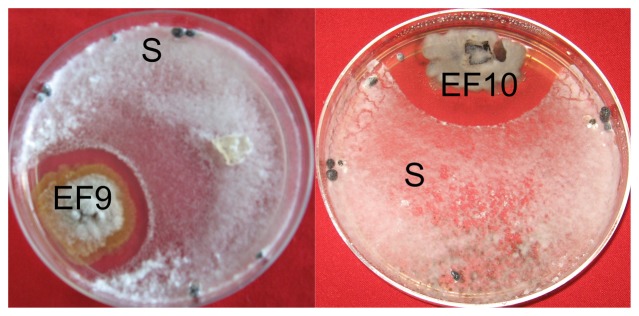
Dual culture bioassay of *Jatropha* endophytic fungi against *Sclerotinia* (S).

Dual culture bioassay of the isolated endophytic fungi of *J. curcas* was carried out to assess their activity against plant pathogenic fungi *R. solani*, *S. sclerotiorum*, and *F. oxysporum*. All the isolates of the *C. truncatum* (EF9, EF10, EF13 and EF14) and *Chaetomium* isolate EF12 showed antagonistic activity against *S. sclerotiorum* and *F. oxysporum* (Table 1). While *N. oryzae* isolate EF8 was effective against *F. oxysporum*, and *G. cammillae* isolate EF15 was effective against *S. sclerotiorum*, none of the endophytic fungi were found effective against *R. solani*. Figure 1 shows the effect of endophytic fungi EF9 and EF10 against *S. sclerotiorum*. On the basis of these observations EF9, EF10, EF12 and EF13 were further taken up for batch culture fermentation and metabolite extraction. Batch culture fermentation was done by culturing each one of these endohytic fungi in one flask of 1 liter capacity containing 300 ml of the wickerham medium.

### Extraction of metabolites from active endophytic fungi

Extraction of metabolites from endophytic fungi EF9, EF10, EF12, EF13, and EF15 was done with ethyl acetate partitioning. Endophytic fungi grown in the liquid culture for 4 weeks were ground after overnight soaking in ethyl acetate and filtered. The filtrate was partitioned with ethyl acetate and dried under vacuum evaporator. After extraction with ethyl acetate, residue was further extracted with butanol. The dried ethyl acetate extract was further partitioned between hexane and 90% methanol. Yield of the extracts in respective solvents is given in Table 2.

**Table 2:** Yield of the different extracts obtained from endophytic fungi isolated from *J. curcas*.

Sl. no.	Endophytic fungi	Yield of different extracts (mg) in 1.2 litre of the media			
		Ethyl acetate	Methanol	Hexane	Butanol
1	EF 9	750	262	142	162
2	EF 10	590	322	149	233
3	EF 12	561	290	130	341
4	EF 13	321	253	63	1055
5	EF 15	501	254	84	173

### Hexane extract of *C. truncatum*

**Figure 2:**
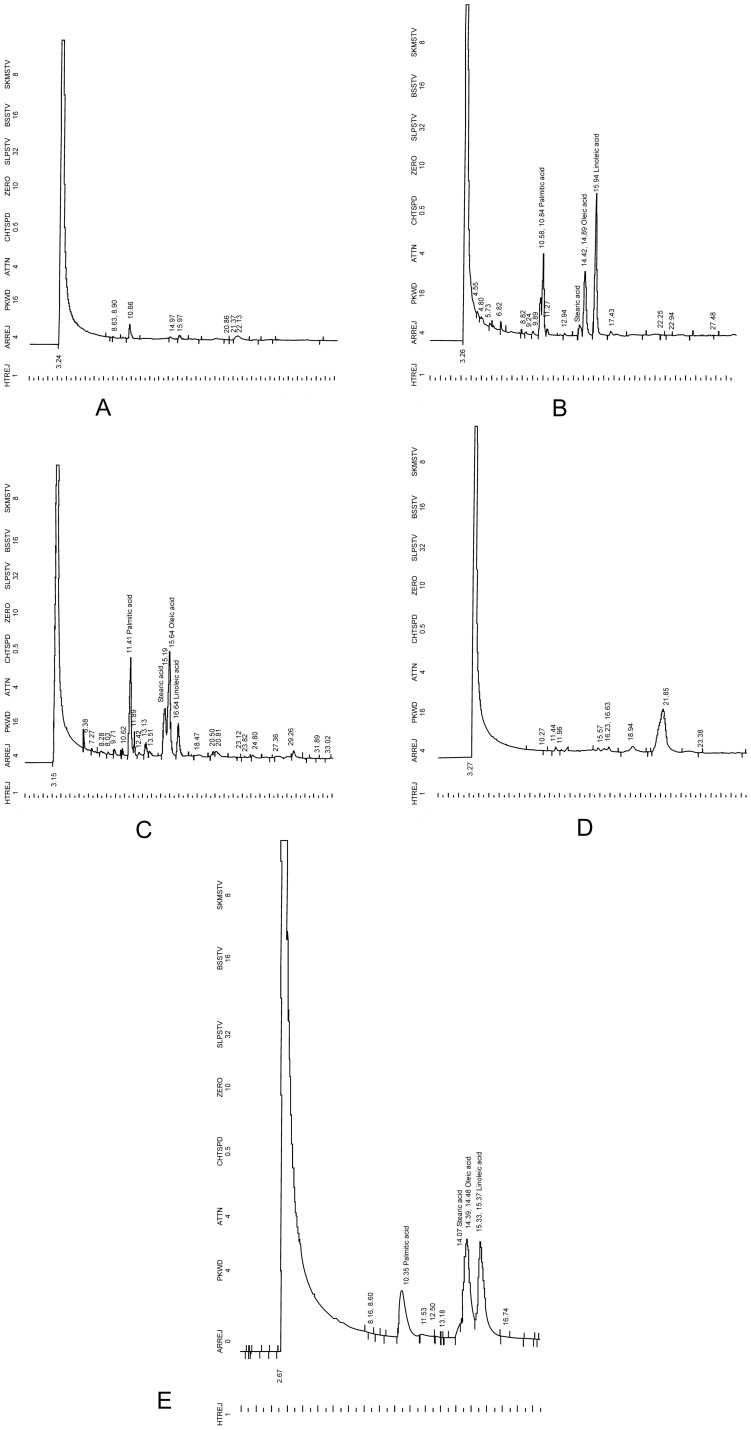
Gas chromatograms of fatty acid composition of hexane extract of endophytic fungi EF 9 (A), EF 10 (B), EF 12 (C) and EF 13 (D) isolated from *J. curcas* and jatropha seed oil (E).

Hexane extracts of *C. truncatum* isolates EF9, EF10, and EF13 yielded an oil and the oil from EF10 was found to be similar to oil of the host plant, i.e., *J. curcas* in fatty acid profile. Variation in the oil yield was recorded; EF10 produced the highest amount of oil, i.e., 99.3 mg/l of media while EF9 and EF13 produced 94.6 mg/l and 42 mg/l under un-optimized conditions. Chromatograms of the oils analyzed by gas chromatography are given in Figure 2 (A, B, C, D, E) and the fatty acid composition is given in Table 3. Fatty acid profile of hexane extract of EF10 was found similar to fatty acid profile of Jatropha seed oil. Jatropha seed oil is known to contain four major fatty acids namely; palmitic acid, stearic acid, oleic acid, and linoleic acid and these four fatty acids were also present in the hexane extract of endophytic fungus EF10, whereas EF12 has few more peaks which are in addition to the peaks of these four fatty acids. Chromatogram of EF9 and EF13 did not have the characteristics peaks of Jatropha seed oil, as they are having only one major peak with some smaller peaks.

**Table 3:** Fatty acid profile of oil isolated from endophytic fungi and Jatropha seed oil.

Source of oil	Percent of fatty acids			
	Palmitic acid	Stearic acid	Oleic acid	Linoleic acid
EF9	43.87	4.75	12.57	36.14
EF10	27.8	3.56	21.6	38.47
EF12*	23.32	13.87	32.04	8.63
Jatropha seed	18.05	2.31	41.87	37.11

Note:

* EF12 has some other peaks too along with these fatty acids.

### Bioassay of the extracts

**Figure 3:**
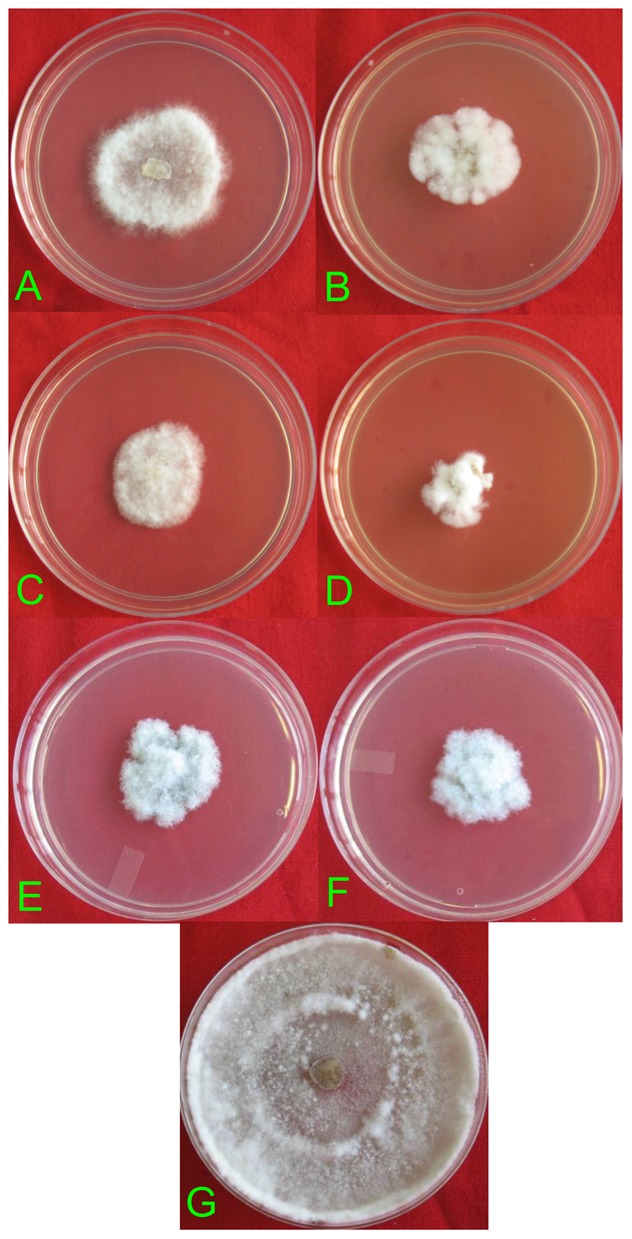
Antifungal activity of solvent extracts of *J. curcas* endophytic fungi against *Sclerotinia sclerotirum*. A- EF9 EtoAc 500 µg/ml, B- EF9 EtoAc 1000 µg/ml, C- EF10 EtoAc 500 µg/ml, D- EF10 EtoAc 1000 µg/ml, E- EF13 EtoAc 500 µg/ml, F- EF13 EtoAc 1000 µg/ml, G- check plate of *S. sclerotiorum*.

Among the four *C. truncatum* isolates, two isolates, viz., *C. truncatum* EF9 and *C. truncatum* EF10 were compared for their activity. EF10 recorded a better GI value of 71.8% compared to 58.4% GI with EF9 at concentration of 500 µg/ml on the 4^th^ day (Table 4). Therefore, for further sub-fractionation *C. truncatum* isolates EF10 and EF13 were selected along with *Chaetomium* sp EF12. Hexane, butanol, and methanol extracts of EF10, EF12, and EF13 were tested at 250 µg/ml and 500 µg/ml for their antifungal activity against *S. sclerotiorum*. Hexane extracts of these fungi were least effective and butanol extract of EF13 showed 66.6% GI on the 4^th^ day, while EF12 has a GI of 10% only (Table 5). Methanol extract of *C. truncatum* isolate EF13 exhibited the highest activity with 83.33% and 66.67% fungal growth inhibition at 500 µg/ml and 250 µg/ml, respectively. Statistically, no significant difference in the GI was observed. Methanol extract of *C. truncatum* isolate EF10 showed 70% GI at 500 µg/ml and 3.46% only at 250 µg/ml. While methanol extract of *Chaetomium* sp. isolate EF12 exhibited moderate activity with 43.33% of GI (Table 6). No growth inhibition was observed in hexane extracts of EF10, EF12, and EF13 at 250 µg/ml; hexane extract of EF10 at 500 µg/ml; butanol extracts of EF10, EF12 and EF13 at 250 µg/ml; butanol extract of EF10 at 500 µg/ml; and methanol extract of EF12 at 250 µg/ml. Photographs of effect of extracts of endophytic fungi on *S. sclerotiorum* is given in Figure 3.

**Table 4:** Effect of ethyl acetate extracts of endophytic fungi of *Jatropha curcas* on growth of *Sclerotinia sclerotiorum*.

Sl. no.	Extract	Per cent Growth inhibition (mean± Standard error)	
		500 µg/ml	1000 µg/ml
1	EF 9	58.4±0.8	74.1±0.7
2	EF 10	71.8±0.4	78.8±2.9

Note: CV 14.1%, CD_0.05_ 10.5.

**Table 5:** Effect of butanol extracts of endophytic fungi of *Jatropha curcas* on growth of *Sclerotinia sclerotiorum*.

Sl. no.	Extract	Per cent Growth inhibition (mean± Standard error)	
		250 µg/ml	500 µg/ml
1	EF 10	0	0
2	EF 12	0	10±5.7
3	EF 13	0	66.6±6.6

Note: CV 47.4%, CD_0.05_ 11.3.

**Table 6:** Effect of methanol extracts of endophytic fungi of *Jatropha curcas* on growth of *Sclerotinia sclerotiorum*.

Sl. no.	Extract	Per cent Growth inhibition (mean± Standard error)	
		250 µg/ml	500 µg/ml
1	EF 10	3.4±3.6	70±5.7
2	EF 12	0	43.3±13.3
3	EF 13	66.6±6.6	83.3±3.3

Note: CV 47.4%, CD_0.05_ 11.3.

## Discussion

Li et al. [19] isolated 57 strains of endophytic fungi from the roots and stems of Jatropha, among which two strains are antagonistic to *Colletotrichum gloeosporioides*. Later a group in India isolated endophytic fungi from *J. curcas* and reported *Leptosphaeria* sp. as predominant fungus [20]. In our study, we isolated nine endophytic fungi from *J. curcas* leaves which represent six species, viz., *C. truncatum* (4), *N. oryzae* (1), *F. proliferatum* (1), *G. cammillae* (1), *A. destruens* (1), and *Chaetomium* sp. (1) with *C. truncatum* as predominant species. No endophytic fungi emerged from *J. curcas* petiole.

*Colletotrichum truncatum* has worldwide occurrence as a plant pathogen, causing diseases of several plants including soybean [21], broad bean, lentil [22], *Stylosanthes* sp., [23], cowpea [24], *Pisum sativum* [25], urdbean [26], christmas rose [27]. However, it has been reported as an endophyte from *Artimisia* sp. [28]. Despite the pathogenic nature of the fungus, it has been utilized for management of several weeds, including *Sesbania exaltata* a noxious weed of soybean [29]. In our study, we found this fungus as predominant endophyte of *J. curcas*. When tested against plant pathogenic fungi, the isolates of *C. truncatum* EF 9, EF10, and EF14 were found active against *S. sclerotiorum* and *F. oxysporum*. Ethyl acetate extract of EF10 isolate caused higher growth inhibition than EF9, although statistically non-significant. Maximum zone of inhibition was observed with EF10. The higher bioactivity of *C. truncatum* EF10 was also observed in its extracts. All the isolates of *C. truncatum* were found yielding oil, and fatty acid composition of oil isolated from EF10, was similar to Jatropha seed oil. *C. truncatum* isolate EF10 also recorded highest yield, while its methanol extract recorded very good antifungal activity.

Endophytic association of *N. oryzae* is well documented and reported from crop plants, including maize [30], ornamental plant, *Rosa hybrida* [31], fruit plant, banana [32], weed, *Parthenium hysterophorus* [33], *Eucalyptus citriodora* Hook. [34], medicinal plant *Tylophora indica* [17], *Nyctanthes arbor-tristis* [35], and *Crataeva magna* [36]. Extracts from an isolate of *N. arbor-tristis* have been shown to have strong antifungal and antibacterial activity also. It has also been shown to be a pathogen of crop plants causing diseases of rice [37], wheat, sorghum, barley [38]. In present study our isolate of *N. oryzae* was found active against *F. oxysporum*.

*Fusarium proliferatum* has been isolated as an endophyte from the mangrove plant [39], pejibaye (*Bactris gasipaes*) [40], and inner bark tissue of *Dysoxylum binectariferum* [41]. It has also been shown to be a pathogen of crop plants causing head blight of oat [42], stem rot in vanilla [43], crown and root rot in wheat [44]. Endophytic fungi from pejibaye have the potential to be developed as biocontrol agent against plant pathogens. Beauvericin, an antibiotic against bacterial pathogens has been isolated from *Fusarium proliferatum* CECT 20569 grown on wheat; the technique of solid state fermentation [45]. In our study, *F. proliferatum* isolate EF11 did not show any activity, although the fungus was grown under different conditions.

*Guignardia* sp. has been isolated as an endophyte from several host plant including *Kandelia candel*, a mangrove plant [46], *Centella asiatica* [47], *Garcinia* [48], *Heterosmilex japonica* [49], sugarcane [50], *Coffea arabica* [51], *Tryptergium wilfordii* [52], *Spondias mombin* [53], *Musa acuminata* [54], leaves of ericaceous plant [55] and *Undaria pinnatifida* [56]. Our isolate *Guignardia camelliae* EF15 from *J. curcus* showed activity against *S. sclerotiorum*, which is the first report of antifungal activity of the fungus. Ethyl acetate extract of this endophytic isolate also showed moderate level of antifungal activity against *Sclerotinia* disease of chickpea.

*Alternaria destruens* is described from *Cuscuta gronovii* [57]. *Alternaria* sp. have been isolated as an endophyte from several host plants including medicinal plants from the western ghats of India [19], *Deschampsia antarctica* [58], *Bletilla ochracea* [59], *Rosa damascaena* [60], *Azadirachta indica* [61], *Catheranthus roseus* [62], *Polygonum senegalense* [63], mangrove plants [64], and *Gossypium* sp. [65]. *A. destruens* has been reported to have herbicidal activity against Dodder, a parasitic weed and it works well in combination with other herbicides as well [66]. Our *A. destruens* isolate from *Jatropha* did not show any activity against *R. solani, S. sclerotiorum*, and *F. oxysporum*.

*Chaetomium* was isolated as an endophyte from many of the plants including *Cinnamomum camphora* [67], *Cucumis sativus* [68], and *Huperzia serrata* [69]. *Chaetomium* sp. from cucumber showed nematicidal activity when applied as seed treatment. *C. globosum* has been the most common species of all *Chaetomium* sp. and has been reported as an endophyte from several host plants including *Canvalia maritime* [70], *Ipopmea pes-caprae*, *Launea sarmentosa* and *Polycarpaea corymbosa* [71], *Oryza sativa* [72], and *Ginkgo biloba* [73]. Endophytic association of *C. globosum* has also been reported from medicinal plants, viz., *Terminalia arjuna*, *Crataeva magna*, *Azadirachta indica*, and *Holarrhena antidysentrica* [74]. Antagonistic activity of *C. globosum* has been identified against major pathogens of cotton, viz., *Macrophomina phaseolina, Fusarium solani*, and *Rhizoctonia solani*, [75]. As an endophyte from *Tylophora indica*, it showed antifungal activity against *S. sclerotiorum* and *F. oxysporum* [17].

In the present study, the fungi we isolated from *J. curcas* leaves showed activity against *S. sclerotiorum* and *F. oxysporum*, however none exhibited activity against *R. solani*. Highest activity against *S. sclerotiorum* was recorded by *C. truncatum* isolate EF13 and its extract whereas *G. camelliae* isolate EF15 showed highest activity against *F. oxysporum* in dual culture bioassay. *C. truncatum* isolate EF13 and *Guignardia camelliae* isolate EF15 can further be explored for its biocontrol potential against *S. sclerotiorum* and *F. oxysporum* respectively. Their metabolites can also be explored for their potential as fungicide.

There are many reports of the production of metabolites by endophytic fungi, which are also produced by the host plant. For example taxol, a major anticancer drug being produced by *Taxus* sp. has been reported from the endophytic fungus of the plant, *Taxomyces andreanae* [76]. Camptothecin, a potent antineoplastic agent has been produced by endophytic fungi of the inner bark of *Camptotheca acuminata*, a known source of camptothecin [77]. In our experiment we found endophytic fungi producing an oil have a fatty acid profile similar to oil produced by its host *J. curcas*. Although, how this apparent co-metabolism is occurring is currently unknown; it may be due to the sharing of genetic material between these organisms.

### Conclusion

The present study gives evidence that *J. curcas* harbors endophytic fungi of beneficial activity. Results indicate that these fungi may be helping the plants in protecting from pathogenic fungi. Endophytic fungi *C. truncatum* isolate EF13 found active in the study can be explored for its potential as a biocontrol agent against *S. sclerotiorum* and *F. oxysporum*, after studying its pathogenicity on crop plants and other non-target effects, while EF10 can be explored for its oil production capacity.

## Materials and Methods

### Sample collection

Leaf and petiole samples of *Jatropha curcas* were collected from plants grown at TERI, New Delhi, India, during June 2007 to August 2007. Immediately after the collection, plant parts were washed with tap water and processed for isolation of endophytic fungi.

### Isolation of endophytic fungi

Endophytic fungi were isolated from the healthy plants as per the procedure of Kumar et al. [17]. The plant parts — leaves and petioles — were surface sterilized with 70% ethanol for 2 minutes followed by 1% sodium hypochlorite for 3 minutes. Surface sterilized plant parts were dried on sterile blotting sheet and then chopped and transferred to malt agar plates, after taking imprint of dried sterile plant part in a petri-plate containing media. These plates were incubated at 24°C for seven days. Hyphal tips of the developing fungal colonies were transferred to fresh malt agar plates.

### Identification of endophytic fungi

#### Slide preparation

Fungal mycelium was stained with cotton blue and mounted in polyvinyl lactic acid glycerol (PVLG) by heating at 65°C for 2–3 days and observed under light microscope.

#### DNA Isolation and amplification

Fungal genomic DNA was isolated from fresh mycelia scrapped from potato dextrose agar (PDA) plates using the DNeasy plant minikit (Qiagen) according to manufacturers' protocol. DNA amplification by PCR was then performed according to the procedure described earlier by Kumar et al. [17].

Purification of the PCR product was done by Montage® PCR centrifugal filter devices as per the suppliers' protocol. Sequencing of the purified PCR product was carried out at LabIndia (Gurgaon, India) on an automated multicapillary DNA sequencer, ABI Prism 3130xl genetic analyzer (Applied Biosystems, USA).

To identify the isolates, sequences were subjected to the BLAST search with the NCBI database [78]. DNA sequences of representative isolates from this study have been submitted to NCBI GenBank database with accession no **GQ176271** to **GQ176279**.

### Bioassay of endophytic fungi against plant pathogenic fungi

Bioassay of endophytic fungi against plant pathogenic fungi was done by dual culture technique [79]. Potato dextrose agar medium (Himedia) was selected for dual culture as it favours growth of plant pathogenic fungi — *Rhizoctonia solani*, *Sclerotinia sclerotiorum*, and *Fusarium oxysporum*. The cultures were obtained from Indian Type Culture Collection, Indian Agricultural Research Institute, New Delhi. Plant pathogenic fungi and endophytic fungi were inoculated on PDA plate at periphery, opposite to each other. After incubation at 24°C for seven days plates were observed and antagonism was expressed by presence of inhibition zone at the point of interaction.

### Batch culture fermentation of endophytic fungi and extraction of their metabolites

Endophytic fungi showing antagonistic property against plant pathogenic fungi, in dual culture bioassay, were inoculated in Wickerham medium [Malt extract (3 g/l); Yeast extract (3 g/l); Peptone (5 g/l); Glucose (Qualigens)-10 g/l; pH-7.2–7.4] (300 ml in 1 litre conical flask) and incubated at 24°C for 24 days under static culture condition. One flask of medium without any inoculam served as a control.

### Extraction of metabolites of endophytic fungi

**Figure 4:**
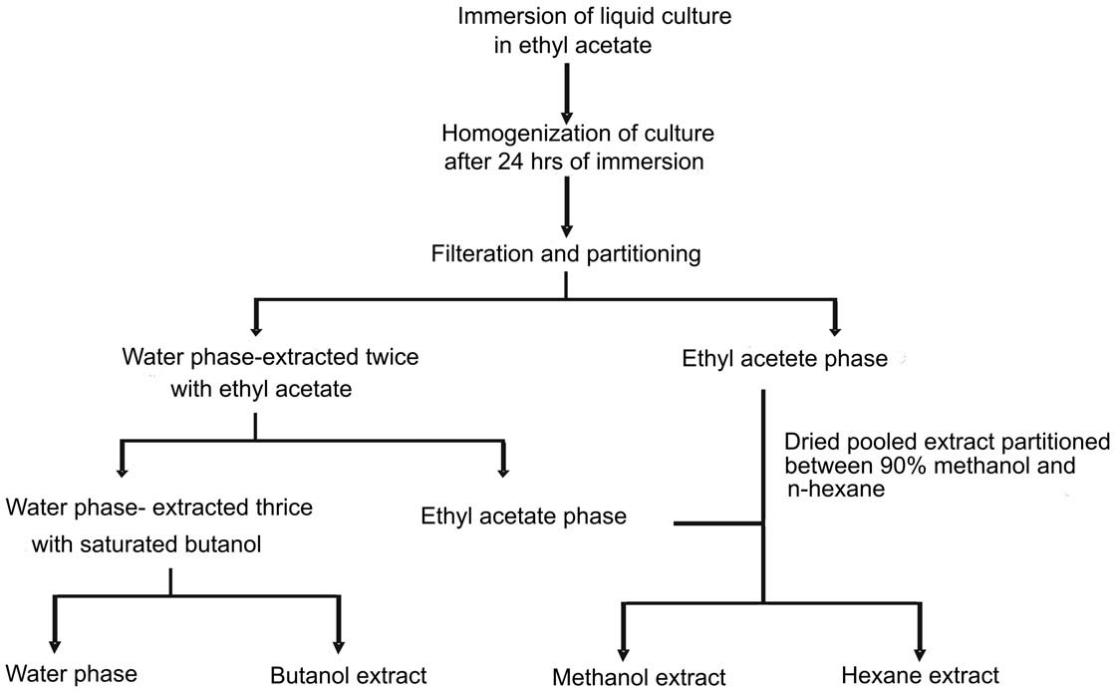
Schematic diagram of extraction procedure for obtaining crude fungal extracts.

After 24 days of incubation, 250 ml of ethyl acetate was added to each flask, mixed, allowed to steep for 24 hrs, blended with a hand blender (INALSA Tech., India) for 15 minutes and filtered by whatman filter paper under vacuum [80]. The filtrate was collected and residual aqueous phase was partitioned thrice with ethyl acetate, followed by butanol (Qualigens, India) partitioning. The extracts were dried with vacuum rotary evaporator (Heidolph Inc, Germany). The ethyl acetate extract was further partitioned between 90% methanol (Qualigens, India) and *n*-hexane (Qualigens, India). The hexane, butanol, and methanol extracts after drying with vacuum rotary evaporator were subjected to further experimentation. Schematic diagram for the extraction of the metabolite has been given in Figure 4.

### Bioassay of extracts of the endophytic fungi

Different extracts were tested against *S. sclerotiorum* by first dissolving 30 mg of dried extract in 800 µl of methanol. From this solution, 200 and 400 µl were added to 30 ml of molten PDA media, mixed and then poured into three 10 cm Petri plates to obtain 250 and 500 µg/ml extract concentrations, respectively. To obtain 1000 µg/ml concentration, 30 mg of dried extracts were dissolved in 400 µl of methanol and 400 µl were added to 30 ml of molten PDA. *S. sclerotiorum* was inoculated at the centre of the plate and radial growth was measured at intervals till the control plate attained the full growth. Control growth plates contained 400 µl of methanol. Per cent growth inhibitions of the extracts were calculated relative to the growth on the control plate.

### Gas chromatography of hexane extract of *C. truncatum* isolates

Gas chromatography (GC) was done to examine the fatty acid composition of the oil extracted from *C. truncatum* and *J. curcas* (host plant). GC was done on Nucon 5700 gas chromatograph equipped with Flame ionisation detector (FID) and capillary column of 30 m length and 0.25 mm ID. Nitrogen was used as carrier gas and hydrogen for flame. Sample preparation was done by mixing 200 µl of oil and 200 µl of methylating reagent (Ethanol∶Benzene∶Acetyl chloride at 20∶4∶1) followed by heating at 72°C for 1 hr and then partitioning with 200 µl of hexane. 2 µl of the hexane soluble part was injected to GC and allowed to run for 20 minute with starting temperature of 180°C and final temp of 232°C with increase of 4°C/minute.

### Data analysis

Growth inhibition (GI) was calculated as per the following formula:
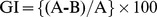
where A = radial diameter of fungus growing on the control plate

B = radial diameter of fungus growing on the experimental plate

All experiments were conducted in triplicate and GI for each replicate was calculated. Analysis of variance of the GI was performed by online statistical package (Web Agri Stat Package-WASP1) of ICAR-Goa regional centre, Goa, India. Least significant difference (LSD) and standard error were calculated and means were compared.
